# Combination therapy of disseminated coccidioidomycosis with caspofungin and fluconazole

**DOI:** 10.1186/1471-2334-6-26

**Published:** 2006-02-15

**Authors:** Dae Won Park, Jang Wook Sohn, Hee Jin Cheong, Woo Joo Kim, Min Ja Kim, Je Hyeong Kim, Chol Shin

**Affiliations:** 1 Division of Infectious Diseases, Department of Internal Medicine, Korea University College of Medicine, 126-1, Anam-dong 5th Str., Seongbuk-gu, Seoul 136–705, Republic of Korea; 2 Division of Pulmonary and Critical Care Medicine, Department of Internal Medicine, Korea University College of Medicine, Seoul, Republic of Korea

## Abstract

**Background:**

The current recommended therapy for diffuse coccidioidal pneumonia involves initial treatment with amphotericin B deoxycholate or high-dose fluconazole, followed by an azole after clinical improvement. Amphotericin B is more frequently used as initial therapy if the patient's deterioration is rapid.

**Case presentation:**

A 31-year-old Korean male with coccidioidomycosis presented to the hospital with miliary infiltrates on chest X-ray (CXR) and skin rash on the face and trunk. Initially, the patient did not respond to amphotericin B deoxycholate therapy. However, following caspofungin and fluconazole combination therapy, the patient showed favourable radiological, serological, and clinical response.

**Conclusion:**

This appears to be the first case of diffuse coccidioidal pneumonia with skin involvement in an immunocompetent patient who was treated successfully with caspofungin and fluconazole. Combination therapy with caspofungin and fluconazole may, therefore, be an alternative treatment for diffuse coccidioidal pneumonia that does not respond to amphotericin B deoxycholate therapy.

## Background

Coccidioidomycosis is an infection caused by inhalation of dimorphic fungi of the genus *Coccidioides *(*C. immitis *and *C. posadasii*). It is endemic in desert regions of the southwestern United States, Central America and South America. This infection has protean manifestations and is frequently misdiagnosed, especially in persons who travel to endemic areas and return to locations where the disease is not typically encountered [1].

Diffuse coccidioidal pneumonia is an unusual complication of a primary pulmonary infection in an immunocompetent patient. It has been associated with a high mortality [1,2]. Diffuse bilateral pulmonary infiltrates may be a result of a hematogenous spread. They may also be a result of a multiple foci of infection secondary to high innoculum exposure. In either case, even early infections are regarded as serious enough to warrant therapy [3]. The recommended therapy for diffuse coccidioidal pneumonia has been amphotericin B, followed by an oral azole antifungal after clinical improvement [4,5]. Recently, updated guidelines recommend high-dose fluconazole or amphotericin B for the treatment of diffuse coccidioidal pneumonia [6]. Although amphotericin B is frequently used as initial therapy, the disease remains difficult to treat and is often characterized by frequent treatment failure and disease relapse. Newly available antifungal agents include voriconazole, posaconazole and caspofungin, each of which may play a role in the treatment of refractory coccidioidosis [4].

Here, we present the case of an immunocompetent patient with diffuse coccidioidal pneumonia who responded to caspofungin and fluconazole therapy without experiencing any adverse effects.

## Case presentation

On October 6, 2004, a 31-year-old Korean male was admitted to the hospital with a fever, chills, general fatigue, cough, dyspnea, night sweats, and a weight loss of 2 kg, all of which he had experienced over the previous four weeks. The patient had smoked half a packet of cigarettes per day for the past 10 years and had been diagnosed four years previously with adrenoleukodystrophy. During August and September of 2004, the patient travelled to Corona, California. The symptoms began while at this location, and he was subsequently treated for bacterial pneumonia at an area hospital. However, his symptoms worsened rapidly and he returned to Korea.

**Figure 1:**
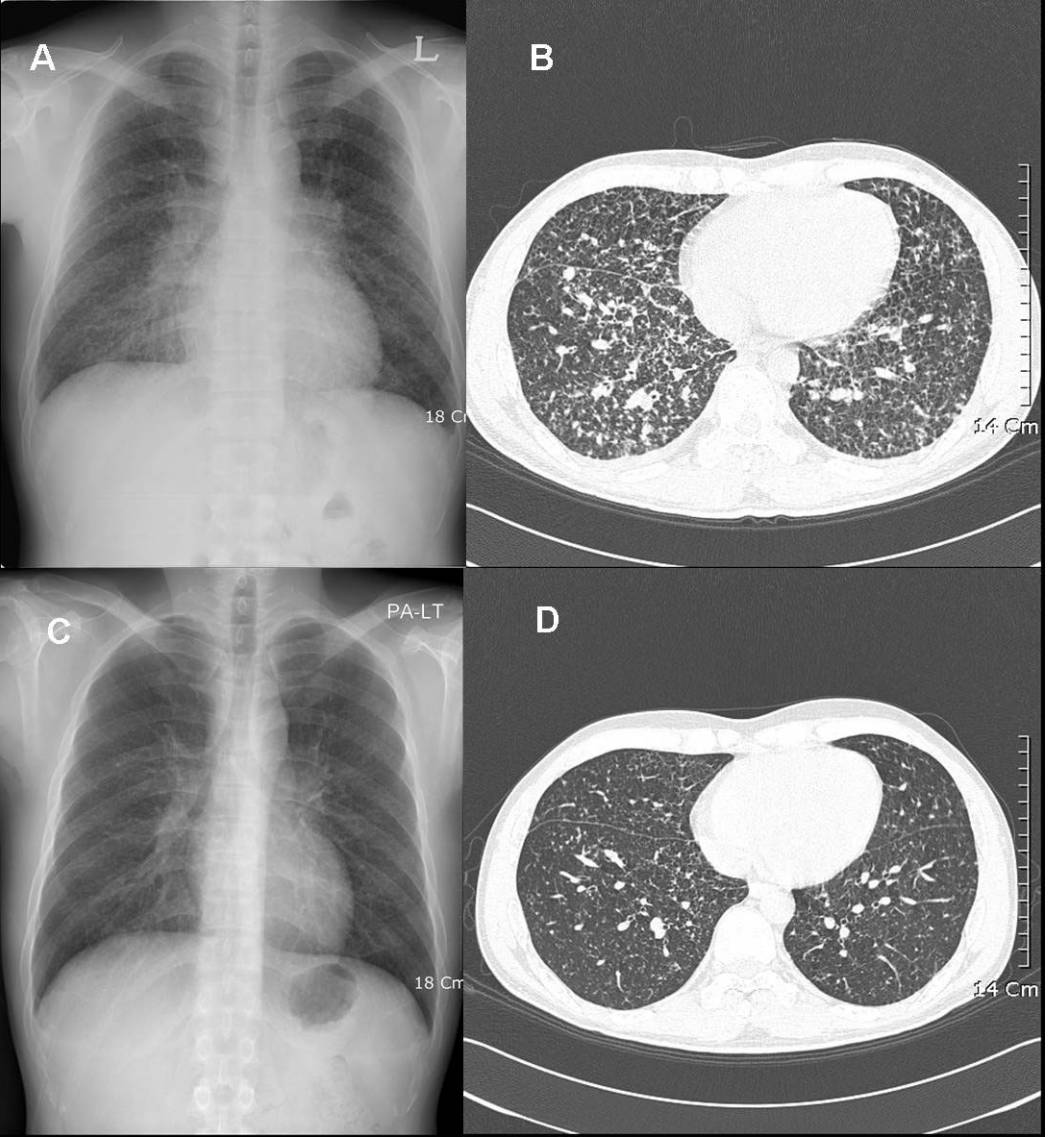
**Chest X-ray (CXR) and high-resolution computed tomography (CT) findings on admission, and again at five months following combined treatment. Top **(on admission): (A) Posterior-anterior chest radiograph (P-A CXR) shows multiple tiny reticular nodules on both lungs; (B) Tiny centrilobular and subpleural nodules are evident in both lungs on the chest CT. **Bottom **(five months after combined treatment): (C) A follow-up CXR shows improvement in the multiple reticular nodules; (D) A follow-up CT scan reveals the improvement of the residual nodules.

On admission to the hospital, the following vital signs were observed: temperature, 37.8°C; blood pressure, 130/70 mmHg; pulse, 92 beats/min; and respiratory rate, 24 breaths/min. A clinical examination revealed a moderately ill-appearing male with multiple, encrusted, erythematous, papular nodules on the face and trunk. There were no other remarkable findings on examination. Laboratory tests revealed leukocytosis with eosinophilia (white blood cell count 23,460 cells/μl; eosinophils 22%), an increased erythrocyte sedimentation rate (100 mm/h), and increased levels of C-reactive protein (24.7 mg/dl). A chest X-ray (CXR) and computerized tomography (CT) scan revealed tiny, multiple nodules in both lung fields (Figure 1, top). Sputum acid-fast bacillus stain and tuberculosis culture were both negative. The patient underwent a bronchoscopy and a skin punch biopsy. The biopsy revealed the presence of a chronic granuloma with thick-walled, mature spherules containing endospores. A subsequent skin tissue culture grew *Coccidioides immitis*. The patient's serum complement fixation (CF) titre was 1:16, and a serum coccidioidal immunodiffusion test for IgG antibody was negative. (All coccidioidal CF antibody testing was carried out at the laboratory of Lynn A. Cheryk, Mayo Clinic, Minnesota, USA). Cerebrospinal fluid (CSF) showed no evidence of meningitis.

The patient was diagnosed with diffuse coccidioidal pneumonia with skin involvement and was treated with amphotericin B deoxycholate (total dosage, 1970 mg; ~1 mg/kg) for 40 days (day five to day 45 of hospitalization). However, the patient still complained of night sweats and a cough. Physical examination revealed a persistent fever (temperature of 38.6–39°C) and no improvement of the skin rash. His general condition progressively deteriorated. The patient developed a right pleural effusion and miliary shadows on the CXR did not improve. A follow-up CF titre performed on day 27 of hospitalization had increased to 1:64. On day 45 of hospitalization, the therapy was changed to caspofungin (initially 70 mg and then 50 mg/day, given intravenously) and fluconazole (400 mg/day, given orally). Sixteen days after the combination treatment (day 60 of hospitalization), a CXR revealed a reduction in both the miliary nodes and the pleural effusion. The patient improved initially, developing only occasional fevers. Forty-nine days after the combination therapy, the patient's peak body temperature fell below 38°C, and the dyspnea had subsided. The serum CF antibody titre had decreased to 1:8. However, the cough remained. By day 125 of the combination treatment, the patient had defervesced, and all of his respiratory symptoms subsided. Although some skin lesions still remained on his face, most were nearly resolved. The serum CF antibody titre was undetectable, and on day 131, caspofungin therapy was discontinued. The patient was subsequently treated with only fluconazole at a dose of 400 mg/day. There were no drug-related side effects noted from the use of the caspofungin and fluconazole. On day 194, his follow-up CF antibody titre increased to 1:256. However, the patient did not experience respiratory symptoms, develop a skin rash, or develop abnormalities on CXR. A follow-up examination five months following completion of the combination treatment revealed minimal residual nodules visible on CXR and CT scan (Figure 1, bottom). The patient's CF antibody titre was 1:128. As of September 2005, the patient was clinically healthy and without any respiratory symptoms and/or fevers. However, he is still receiving suppressive treatment with fluconazole.

## Discussion

In 2000, the Infectious Diseases Society of America (IDSA) published guidelines for the treatment of coccidioidomycosis [5]. Amphotericin B was recommended as first-line therapy for diffuse pneumonia caused by *Coccidioides *organisms. Despite the use of amphotericin B therapy, diffuse coccidioidal pneumonia remains difficult to treat, and a patient's course is often characterized by frequent treatment failure and disease relapse.

Recently, a number of new antifungal agents have been evaluated for their activity against *C. immitis*, in both *in vitro *and *in vivo *studies. Agents that have been shown to be potentially useful in the treatment of coccidioidomycosis include voriconazole, caspofungin, and posaconazole [4,7-9].

Lutz et al. [9] published *in vitro *data for posaconazole showing fair fungicidal activity against *C. immitis*. They also noted that posaconazole showed superior activity to fluconazole or itraconazole in a murine model of disseminated non-meningeal coccidioidomycosis. Anstead et al. [7] reported a successful outcome in six patients treated with posaconazole who were refractory to refractory to conventional antifungal therapies. Another recent report described the successful treatment with voriconazole in a human with disseminated non-meningeal coccidioidomycosis [8]. However, both posaconazole and voriconazole were not available for treatment of this particular patient.

Caspofungin is the first of a new class of antifungal agents, the echinocandins, which target the fungal cell wall itself via interruption of the β-(1,3)-D-glucan synthesis pathway [10,11]. This mechanism of action is different from that of amphotericin B and azoles. β-(1,3)-D-glucan synthase is absent from mammalian cells. Caspofungin has received US Food and Drug Administration approval for the treatment of mucosal and invasive candidiasis, and also for salvage therapy of invasive aspergillosis [10]. Caspofungin was therapeutically effective in a murine model of *C. immitis *infection [12], despite its limited *in vitro *activity against the fungus when minimum inhibitory concentrations (MICs) were determined. However, an improved correlation between *in vivo *activity and the minimum effective concentration (MEC) – the lowest dose of drug that causes morphologic effects on fungal hyphae – was observed [12]. In this study, mice infected with one of two strains of *C. immitis *(each with an MEC of 0.125 mg/mL but one with an MIC of 8 mg/mL and the other with an MIC of 64 mg/L) responded equally well to treatment with caspofungin. One recent report describes the successful treatment of disseminated coccidioidomycosis in a renal transplant recipient with caspofungin [13]. Furthermore, another report describes a coccidioidal meningitis treatment failure in which caspofungin was not detected in CSF, even though it was found to be above MIC in his serum [14]. We think that the limited CSF penetration of caspofungin may be due to the large molecular mass, water solubility, and high protein binding of caspofungin [10].

Amphotericin B therapy failed to prevent disease progression in our patient. At the time of initial treatment, the recommended regime consisted of treatment with amphotericin B, followed by an azole if clinical improvement was achieved [4,5]. The only new antifungal agent available to us was caspofungin. Since clinical and *in vitro *data for caspofungin were limited, caspofungin monotherapy could not be selected because of the severity of the illness. The scarcity of evidence of *in vitro *antagonism when combined with other antifungal agents led us to consider combination therapy [10]. We considered fluconazole or itraconazole as a combination antifungal with caspofungin. We chose fluconazole because itraconazole has absorption problems and we would be unable to measure serum levels of itraconazole. Combined treatment against *C. immitis *is currently not recommended, and the possibility of antagonism or synergism between caspofungin and fluconazole has not yet been fully evaluated. However, an *in vitro *study reported that although synergy was low between caspofungin and fluconazole, synergism was the pattern most often observed [15]. The mechanisms proposed for its synergy may be simultaneous inhibition of different fungal cell targets, such as the cell wall and membrane targets.

This data, the limited treatment options, and the severity of the illness prompted us to carry out a trial of combined therapy with caspofungin and fluconazole. At the time of this report, the updated IDSA guidelines published in September 2005 recommended amphotericin B or high-dose fluconazole for the treatment of diffuse coccidioidal pneumonia [6]. The high-dose fluconazole therapy was not recommended when our patient was treated.

On day 16 after initiating combined therapy with caspofungin and fluconazole, a CXR revealed a reduction in both the multiple tiny reticular nodules and the pleural effusion. The patient began to improve clinically, even though the CF antibody titre increased from an undetectable level to 1:128 after completion of combined therapy. The CF titre is considered a prognostic factor. However, our patient was clinically healthy: the skin rash had subsided, and the chest CT showed improvement. Thus, we did not perform any further evaluations. An initial bone scan and CSF analysis showed no abnormalities. The patient did not complain of a headache or localized pain. Over the course of this study, the patient was carefully monitored, and he will continue to receive fluconazole for the foreseeable future.

Combination therapy with caspofungin and fluconazole may be an alternative treatment for diffuse coccidioidal pneumonia with non-meningeal extrapulmonary involvement that is unresponsive to amphotericin B therapy. However, determining the best course of therapy remains a challenge. Further clinical studies evaluating this combination are required.

## Conclusion

Although combined therapy with caspofungin and fluconazole against *C. immitis *is not currently recommended, it may represent an alternative treatment for cases that do not respond to amphotericin B deoxycholate.

## Competing interests

The author(s) declare that they have no competing interests.

## Authors' contributions

HJC, WJK, and MJK advised on the management of the patient and assisted in editing the manuscript. JHK and CS performed the bronchoscopic biopsy and provided clinical details. DWP and JWS drafted the manuscript and were involved in patient management and follow-up. All authors read and approved the final manuscript.

## Pre-publication history

The pre-publication history for this paper can be accessed here:


